# Emerging a Novel VOPP1-EGFR Fusion Coexistent With T790M as an Acquired Resistance Mechanism to Prior Icotinib and Sensitive to Osimertinib in a Patient With EGFR L858R Lung Adenocarcinoma: A Case Report

**DOI:** 10.3389/fonc.2021.720819

**Published:** 2021-12-22

**Authors:** Xia Wang, Weiwei Peng, Zhimin Zeng, Jing Cai, Anwen Liu

**Affiliations:** ^1^ Department of Oncology, The Second Affiliated Hospital of Nanchang University, Nanchang, China; ^2^ Department of Oncology, People’s Hospital of Ganzhou, Ganzhou, China

**Keywords:** EGFR fusion, lung adenocarcinoma, icotinib, osimertinib, VOPP1

## Abstract

**Background:**

Epidermal growth factor receptor (EGFR) fusions are rare genomic events in non-small-cell lung cancer (NSCLC). Clinical support and evidence to guide management are absent for NSCLC patients harboring EGFR fusion.

**Case Presentation:**

In this case report, we describe a 69-year-old female who received right lobectomy and was diagnosed with pathological stage IIIA lung adenocarcinoma harboring EGFR L858R. Twenty months later he had recurrent disease in the liver, lung, and bone, and was treated with icotinib. A novel vesicular overexpressed in cancer pro-survival protein 1 (VOPP1)-EGFR fusion gene coexistent with T790M were identified by next-generation sequencing using pericardial effusion and blood samples after icotinib treatment, which led to progression after icotinib six months and suggested a potential resistance mechanism. Subsequently, the patient was switched to osimertinib treatment, which resulted in a progression-free survival interval of more than 11 months.

**Conclusions:**

The present results suggested that acquired VOPP1-EGFR fusion gene with T790M potentially serve an additional resistance mechanism to first-generation EGFR tyrosine kinase inhibitors in EGFR-mutated NSCLC. And the present case increases the evidence supporting use of osimertinib for treatment of NSCLC patients harboring EGFR fusion.

## Introduction

Epidermal growth factor receptor (EGFR) fusions are rare genomic events in non-small-cell lung cancer (NSCLC) (1–4). EGFR fusions were found at a frequency of 0.13% in the MSK-IMPACT NSCLC data (4). A total of eight EGFR fusion partner genes have been reported, including RAD51, TNS3, SEPTIN14, ZCCHC6, SHC1, KIF5B, FGFR1 and PURB, in NSCLC (1, 2, 4–9). There are no standard treatment options for NSCLC patients harboring an EGFR fusion.

Although the efficacy of EGFR tyrosine kinase inhibitors (TKIs) is significant in NSCLC patients harboring EGFR sensitive mutations, patients will inevitably develop acquired drug resistance to EGFR-TKIs after 10-14 months of 1^st-^ or 2^nd^-generation TKIs treatment (10, 11). The most commonly acquired resistance mechanism is EGFR T790M mutation, which accounts for about 50–60% of NSCLC patients with acquired resistance to 1^st^- or 2^nd^-generation EGFR-TKIs (10–13). Recent evidence have suggested that acquisition of gene fusions, including EGFR fusion, are possible rare cause of acquired resistance to targeted therapies in NSCLC (7, 14–17).

Herein, we report the first case involving an NSCLC patient with an unreported emerging vesicular overexpressed in cancer pro-survival protein 1 (VOPP1)-EGFR fusion gene who experienced a durable antitumor response to osimertinib after acquired resistance to icotinib.

## Case Description

**Figure 1:**
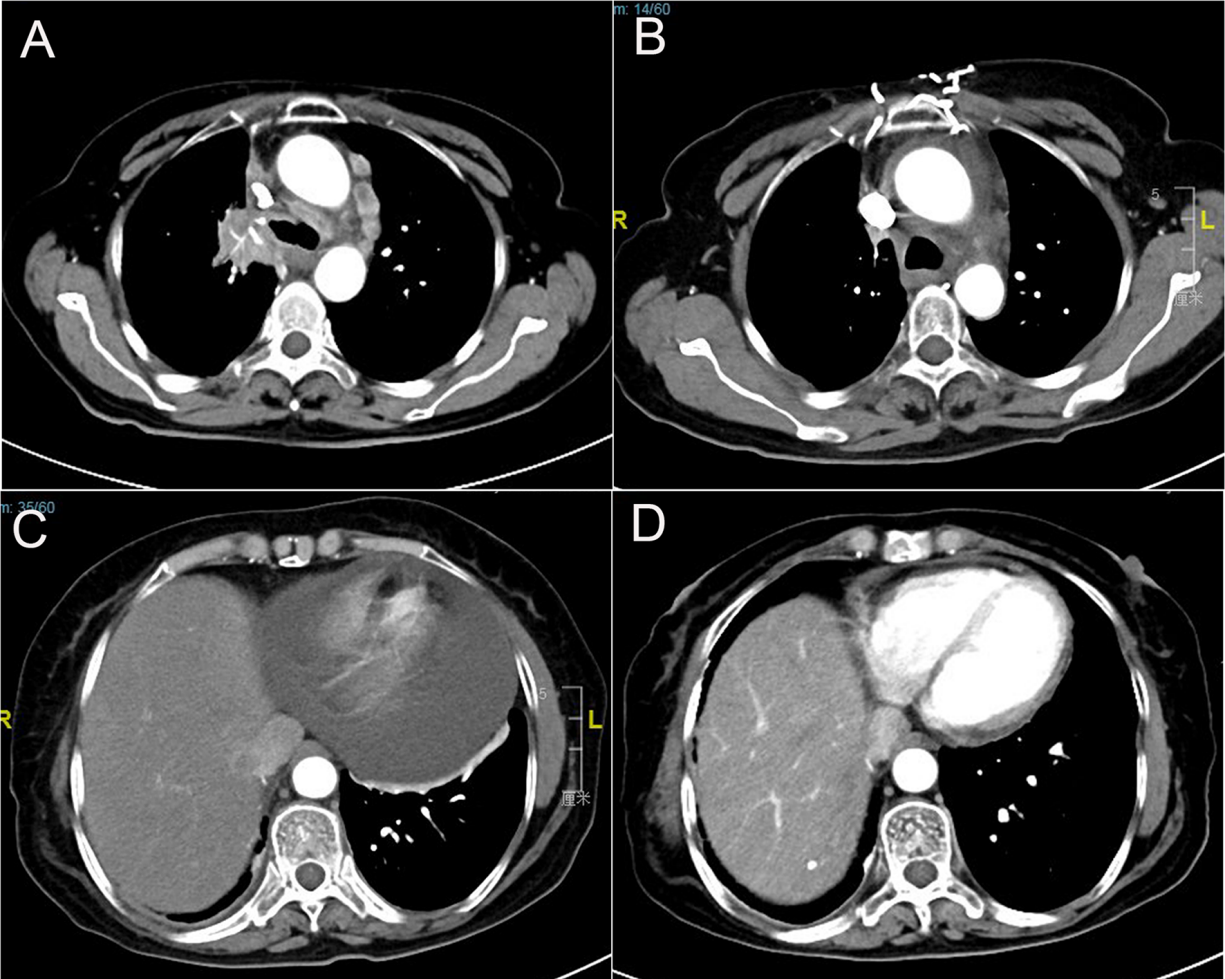
Computed tomography scans show **(A)** lung lesions and mediastinal lymph node metastasis before icotinib therapy; **(B)** partial response in lung lesions after icotinib treatment; **(C)** pericardial effusion development; and **(D)** pericardial effusion was well controlled after osimertinib treatment.

**Figure 2:**
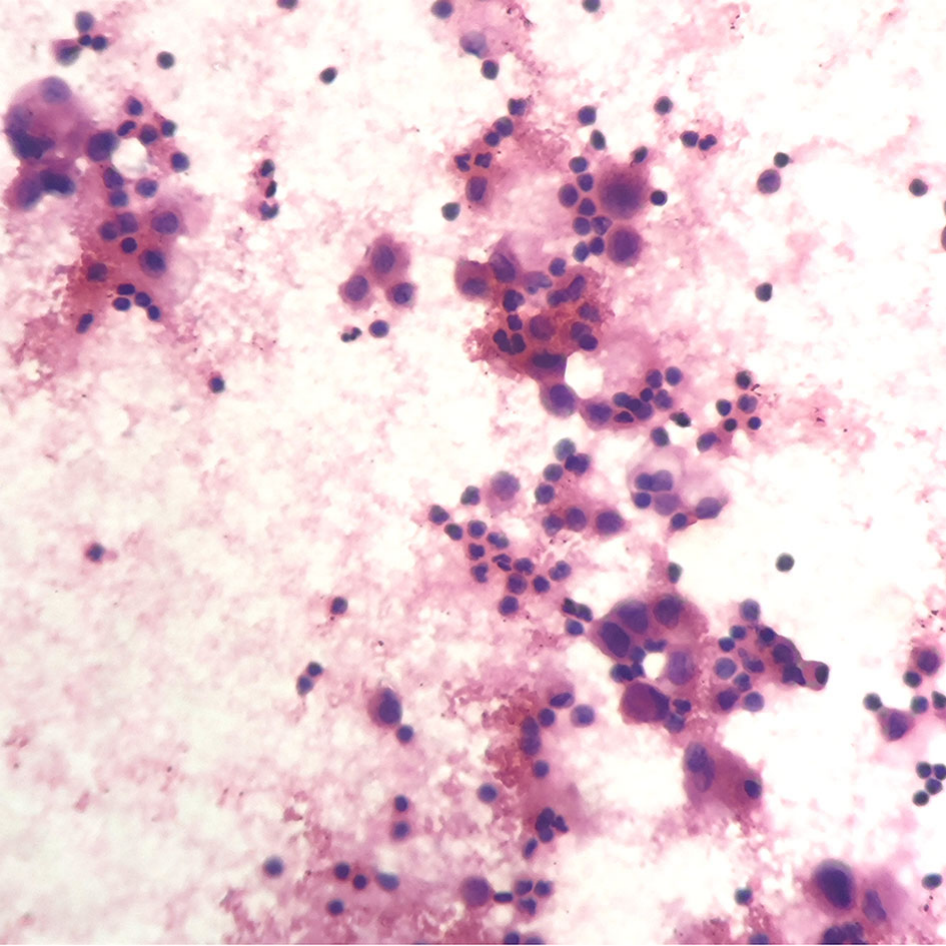
Pericardiocentesis indicated positive cytology of pericardial effusion (hematoxylin-eosin stain; original magnification ×400).

**Figure 3:**
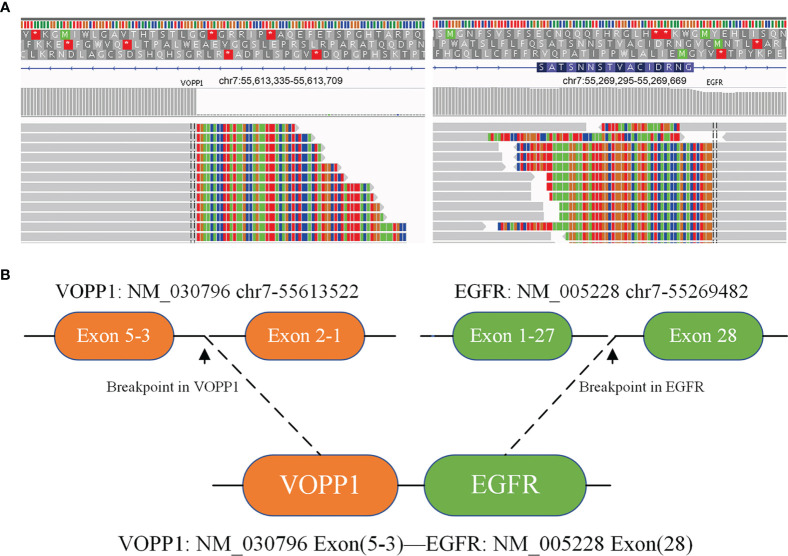
Next-generation sequencing analysis showed VOPP1-EGFR fusion mutations by repeat liquid biopsy of pericardial effusion and blood samples. **(A)** Integrative Genomics Viewer snapshot of VOPP1-EGFR fusion; **(B)** a schematic map of the VOPP1-EGFR fusion protein domain structure *indicates that termination codons cannot encode any amino acids.

A 67-year-old female underwent right lobectomy in October 2017 followed by adjuvant chemotherapy with pemetrexed and cisplatin for 4 cycles for pathological stage IIIA (pT2N2M0) lung adenocarcinoma (moderately differentiated). The tumor tissue was subjected to next-generation sequencing (NGS) (Berry oncology, Fujian China) postoperatively and showed the EGFR exon 21 L858R point mutation (c.2573T>G, p.Leu858Arg). However, liver, lung, and bone metastases appeared twenty months later (**Figure 1A**). Because of the detection of the L858R mutation by liquid biopsy, the patient received icotinib (125 mg three times daily) starting in August 2019, and a partial response was observed. No progression of metastatic foci was identified (**Figure 1B**); however, neoplastic pericardial effusion (**Figure 2**) developed with signs of disease progression after 6 months of icotinib treatment (**Figure 1C**). A repeat liquid biopsy of pericardial effusion and blood samples was performed for NGS to explore the mechanism(s) of acquired resistance and showed an emerging acquired VOPP1-EGFR fusion gene coexistent with T790M (**Figure 3**). Subsequently, the patient was switched to osimertinib (80 mg daily) in February 2020. The metastatic lesions were well controlled in liver, lung and bone after osimertinib treatment. Chest computed tomography showed a small pericardial effusion after osimertinib treatment and the patient achieved a progression-free survival interval of more than 11 months (**Figure 1D**). The timeline of the clinical course is summarized in **Figure S1**.

## Discussion

EGFR fusions, which are likely to behave as oncogenic drivers, are rare genomic events in NSCLC (1–4). A seminal paper reported EGFR fusions in 5 cases (0.05%) in lung cancer, most commonly EGFR-RAD51 fusions, four of whom were treated with erlotinib (1). Kartik et al. also demonstrated that EGFR-RAD51 fusions are oncogenic in preclinical studies and is able to activate downstream tumorigenic signaling *via* the MAPK and PI3K/AKT pathways (1). As an anti-apoptotic protein, VOPP1 overexpression has been observed in multiple malignancies such as hepatocellular carcinoma, glioblastoma and breast cancers (18–20). VOPP1 was one of the most recurrent partners involved in potential oncogenic fusions in glioblastoma (20). There is currently no report on VOPP1 fusion in primary lung adenocarcinoma.

Due to the low incidence and the lack of detection technology, the current understanding of EGFR fusions is not sufficient. In this study, we report for the first time a patient with NSCLC with a novel VOPP1-EGFR fusion, which was not present prior to icotinib therapy. Fifteen EGFR fusion cases have been reported in NSCLC, most commonly RAD51 (8 of 15 cases) (1, 2, 4–9). Among them, eleven patients received EGFR-TKIs. One patient showed disease stabilization in response to afatinib. The other ten patients achieved a partial response with 1^st-^ or 2^nd^-generation TKIs. This is also the first case report in which an EGFR fusion was sensitive to a 3^rd^ generation EGFR-TKI (osimertinib). Therefore, for advanced NSCLC patients harboring EGFR fusion, EGFR-TKIs may hold promise as the initial treatment.

The most common mechanisms of acquired resistance to EGFR-TKIs include secondary EGFR mutation, bypass of signaling pathway activation and histologic transformation (10, 11). Developments in NGS have created a new method for the detection of a large number of gene events, which expanded our understanding of TKI resistance mechanisms in NSCLC. Recent evidence has suggested that the acquisition of various gene fusions could also mediate the process of acquired resistance to targeted therapies in NSCLC (7, 14–17, 21–26). A case study indicated that the KIF5B-RET fusion gene may underlie the acquisition of resistance to icotinib (14). An EGFR-FGFR1 fusion with apparent T790M dropout was detected after 1^st^ and 3^rd^ generation EGFR TKIs (erlotinib then osimertinib) but was not present prior to EGFR-targeted therapy and was identified as a rare resistance mechanism to EGFR-TKIs (7).

As a study with 43 patients in the Anaplastic Lymphoma Kinase (ALK) fusion-positive cohort, a possible mechanism of resistance was identified in 86% of ALK+ patients (23). RALGAPA1-NRG1 fusion on the post-alectinib tumor sample and CCDC6-RET fusion on the post-brigatinib biopsy were identified as ALK resistance mechanisms. To demonstrate RALGAPA1-NRG1 fusion was both functional and could mediate ALK inhibitor resistance of NSCLC, Clustered Regularly-Interspaced Short Palindromic Repeats were used to engineer this fusion into the H3122 cell line to (H3122-NRG1). The presence of the RALGAPA1-NRG1 fusion in H3122 was confirmed by genomic sequencing. H3122-NRG1 cells showed marked resistance to ALK inhibitor and was sensitive to pan-HER inhibitor afatinib.

Novel fusion events have been identified in 3-10% of cases of acquired resistance to second-line osimertinib, and they can co-occur with EGFR C797S, BRAF mutation and MET amplification (22). With advances in detection methods, some rare gene fusions have been identified as potentially mechanisms of acquired resistance to second-line osimertinib in NSCLC patients, including FGFR3-TACC3, RET-ERC1, CCDC6-RET, NTRK1-TPM3, NCOA4-RET, GOPC-ROS1, AGK-BRAF and ESYT2-BRAF (21, 22, 24–26). FGFR3-TACC3 and CCDC6-RET fusion (1each), coexistent with T790M loss, have been identified in 4.8% (2/41) of cases of acquired resistance to second-line osimertinib (24).

Here, we describe a case of VOPP1-EGFR fusion coexistent with T790M detected by repeat liquid biopsy after progression on icotinib treatment in one patient. Therefore, we propose that this VOPP1-EGFR fusion gene could potentially serve as an additional resistance mechanism to TKIs in NSCLC. However, this acquired EGFR fusion remains to be tested and awaits further research.

In summary, this case indicated that the VOPP1-EGFR fusion gene may underlie the acquisition of resistance against EGFR-TKIs and suggest that rebiopsy (including liquid biopsy) should be routinely performed when disease progresses in patients treated with EGFR-TKIs. The NGS assay provides a powerful tool for identifying rare or atypical genomic mutation events in patients with NSCLC and should be encouraged in clinical practice.

## Data Availability Statement

The original contributions presented in the study are included in the article/**Supplementary Material**. Further inquiries can be directed to the corresponding author.

## Ethics Statement

This report was approved by institutional ethics committee of Second Affiliated Hospital of Nanchang University, Nanchang, China. The patients/participants provided their written informed consent to participate in this study.

## Author Contributions

All authors made substantial contributions to conception and design, acquisition of data, or analysis and interpretation of data; took part in drafting the article or revising it critically for important intellectual content; agreed to submit to the current journal; gave final approval of the version to be published; and agree to be accountable for all aspects of the work.

## Funding

This work was supported by the National Natural Science Foundation [grant number 82060577, 82060547].

## Conflict of Interest

The authors declare that the research was conducted in the absence of any commercial or financial relationships that could be construed as a potential conflict of interest.

## Publisher’s Note

All claims expressed in this article are solely those of the authors and do not necessarily represent those of their affiliated organizations, or those of the publisher, the editors and the reviewers. Any product that may be evaluated in this article, or claim that may be made by its manufacturer, is not guaranteed or endorsed by the publisher.

## Supplementary Material

Supplementary Figure S1 Timeline of the clinical course of the patient with VOPP1-EGFR fusion.
