# Supplementary figures and images for: Emerging a Novel VOPP1-EGFR Fusion Coexistent With T790M as an Acquired Resistance Mechanism to Prior Icotinib and Sensitive to Osimertinib in a Patient With EGFR L858R Lung Adenocarcinoma: A Case Report
Source: Front Oncol. 2021 Dec 22;11:720819. doi: 10.3389/fonc.2021.720819 (PMC8727519; doi:10.3389/fonc.2021.720819)

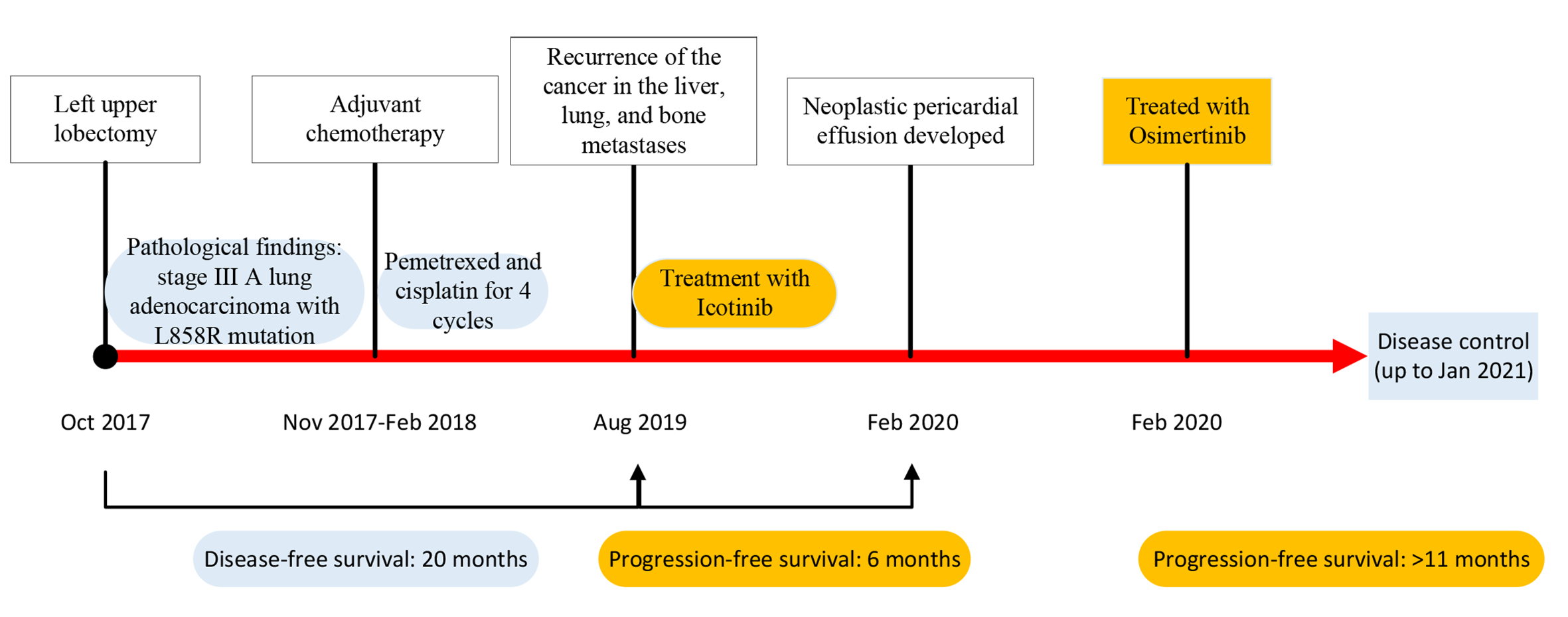

Supplement: Supplementary Figure S1 — Timeline of the clinical course of the patient with VOPP1-EGFR fusion. [file Image_1.tif]
